# Characterization and Functional Analysis of the 17-Beta Hydroxysteroid Dehydrogenase 2 (*hsd17b2*) Gene during Sex Reversal in the Ricefield Eel (*Monopterus albus*)

**DOI:** 10.3390/ijms25169063

**Published:** 2024-08-21

**Authors:** Ruyi Chen, Haoyu Zhu, Xiaoling Zhang, Lingli Li, Jinglin Xu, Zhimin Tan, Jialin Su, Ke Feng, Kaili Chen, Hongyan Xu

**Affiliations:** Integrative Science Center of Germplasm Creation in Western China (CHONGQING) Science City & College of Fisheries, Key Laboratory of Freshwater Fish Reproduction and Development Ministry of Education, Key Laboratory of Aquatic Sciences of Chongqing, Southwest University, Chongqing 402460, China; chenruyiod@gmail.com (R.C.); zzzhy703@163.com (H.Z.); zhangxiaoling0602@163.com (X.Z.); apleuv@163.com (L.L.); xjl070901@163.com (J.X.); 17383082129@163.com (Z.T.); suejialin@163.com (J.S.); fengke127@163.com (K.F.)

**Keywords:** *hsd17b2*, chemical in situ hybridization, sex reversal, hormone induction, ricefield eel

## Abstract

In mammals, 17-beta hydroxysteroid dehydrogenase 2 (Hsd17b2) enzyme specifically catalyzes the oxidation of the C17 hydroxyl group and efficiently regulates the activities of estrogens and androgens to prevent diseases induced by hormone disorders. However, the functions of the *hsd17b2* gene involved in animal sex differentiation are still largely unclear. The ricefield eel (*Monopterus albus*), a protogynous hermaphroditic fish with a small genome size (2*n* = 24), is usually used as an ideal model to study the mechanism of sex differentiation in vertebrates. Therefore, in this study, *hsd17b2* gene cDNA was cloned and its mRNA expression profiles were determined in the ricefield eel. The cloned cDNA fragment of *hsd17b2* was 1230 bp, including an open reading frame of 1107 bp, encoding 368 amino acid residues with conserved catalytic subunits. Moreover, real-time quantitative reverse transcription polymerase chain reaction (RT-qPCR) analysis showed that *hsd17b2* mRNA expressed strongly in the ovaries at early developmental stages, weakly in liver and intestine, and barely in testis and other tissues. In particular, *hsd17b2* mRNA expression was found to peak in ovaries of young fish and ovotestis at the early stage, and eventually declined in gonads from the late ovotestis to testis. Likewise, chemical in situ hybridization results indicated that the *hsd17b2* mRNA signals were primarily detected in the cytoplasm of oogonia and oocytes at stage I–II, subsequently concentrated in the granulosa cells around the oocytes at stage Ⅲ–Ⅳ, but undetectable in mature oocytes and male germ cells. Intriguingly, in ricefield eel ovaries, *hsd17b2* mRNA expression could be significantly reduced by 17β-estradiol (E2) or tamoxifen (17β-estradiol inhibitor, E2I) induction at a low concentration (10 ng/mL) and increased by E2I induction at a high concentration (100 ng/mL). On the other hand, both the melatonin (MT) and flutamide (androgen inhibitor, AI) induction could significantly decrease *hsd17b2* mRNA expression in the ovary of ricefield eel. This study provides a clue for demonstrating the mechanism of sexual differentiation in fish. The findings of our study imply that the *hsd17b2* gene could be a key regulator in sexual differentiation and modulate sex reversal in the ricefield eel and other hermaphroditic fishes.

## 1. Introduction

Short-chain dehydrogenases/reductases (SDRs) are mostly NAD(P)H dependent enzymes, which specially catalyze oxidation/reduction reactions by using the substrates of steroids, retinols, prostaglandins, polyols, and xenobiotics [1]. The 17 beta-hydroxysteroid dehydrogenase (17β-HSD) family is the main component of the SDR superfamily, except for HSD17B5 (Aldo-keto reductases, AKR superfamily) [2]. A series of 17β-HSD enzymes act as the downstream regulators of steroid synthesis and metabolism by catalyzing the steroid-specific conversion of hydroxy and keto groups at position C17 in steroid substrates, delicately regulating transformation among androstenedione, testosterone (T), estrone and estradiol [3,4,5].

As a member of the 17β-HSD family, 17-beta hydroxysteroid dehydrogenase 2 gene (*Hsd17b2*) was firstly characterized from human prostate cDNA library, initiating a cascade of relevant research. In mammalian studies, the *hsd17b2* gene was found to be widely distributed in tissues, especially in the placenta, mammary gland, uterus, and prostate, that are the targeted tissues for sex steroids [6]. Other tissues participating in steroid synthesis or metabolism such as liver and kidney can express *hsd17b2* as well. Recent studies even indicate that diseases in the digestive tract are also closely associated with abnormal expression of *hsd17b2* [7,8]. Functional analysis in humans and rats reveals that *hsd17b2* is the critical steroid downstream element to drive the conversion between active and inactive forms of estrogens and androgens, limiting their active forms in the blood circulation and thus reducing disease risk caused by their abnormal concentration [9]. Notably, in contrast to Hsd17b1 and Hsd17b3 that are highly conserved in evolution, Hsd17b2 varies remarkably in different species and leads to functional diversity. Compared to humans, the Hsd17b2 enzyme in rodents lacks a lysine enrichment zone or the reactive subunit in C-terminal and N-terminal regions, resulting in partial catalytic discrepancy. Moreover, mice overexpressing human *Hsd17b2* are infertile and exhibit growth retardation due to decreased pubertal bone growth [10,11]. Knocking out the *hsd17b2* gene leads to high mortality during embryonic development owing to disturbed placental organization [12]. In non-mammalian vertebrates, the Hsd17b2 enzyme in ruffs is the candidate to specifically convert T to androstenedione that helps to shape social behavior [13]. In teleosts, *hsd17b2* was first identified in zebrafish (*Danio rerio*); its gene structure shared a low identity with mammals, being functionally absent due to a nucleotide replacement in the first exon [14]. In other species, such as tiger puffer (*Takifugu rubripes*), spotted green pufferfish (*Tetraodon nigroviridis*), and bastard halibut (*Paralichthys olivaceus*), the *hsd17b2* gene was also identified [15,16]. In summary, Hsd17b2 specifically catalyzes the oxidation of C17 hydroxyl group and is critical for hormonal steroid metabolism, especially for conversion among estrone, 17β-estradiol (E2), and T, which has been validated in many vertebrates. However, to keep pace with the evolution of steroid hormone nuclear receptors in variant endocrinological conditions, function divergence may eventually occur in different vertebrates. Additionally, the zygote arrest-1 (*zar1*) gene, an oocyte-specific and maternal gene that is mainly distributed in the ovary, is considered as the crucial element for early oogenesis [17]. Here, we used it as a marker gene specifically expressed in the female germ cells to set as a control for analyzing the *hsd17b2* gene expression profile in this study.

The ricefield eel (*Monopterus albus*) is a protogynous hermaphroditic fish that is an economically important freshwater fisheries species in China and is also widely cultured in Southeast Asia. The mechanisms behind the ricefield eel’s sex reversal are still unclear due to the complicated neuroendocrine network affected by various environmental factors, including light, water temperature, feeding conditions, social behaviors, etc. To date, many studies have been conducted in ricefield eels to demonstrate the mechanism of sex reversal from ethological, physiological, biochemical, cytological, and molecular aspects [18,19,20,21]. The sex-reversal mechanisms may be more complicated than we previously appreciated [22,23,24]. To elucidate the molecular regulation network of sex reversal in ricefield eels, many genes associated with sex determination have been characterized [25,26,27], while the genes for downstream steroid synthesis and metabolism have scarcely been studied. Here, we cloned and characterized the *hsd17b2* gene, examined its mRNA expression patterns, analyzed the relationship or interaction between hormones and *hsd17b2* mRNA expression, and elucidated the potential functions of the *hsd17b2* gene during sex reversal in ricefield eels.

## 2. Results

### 2.1. Identification of hsd17b2 cDNA in Ricefield Eel

**Figure 1:**
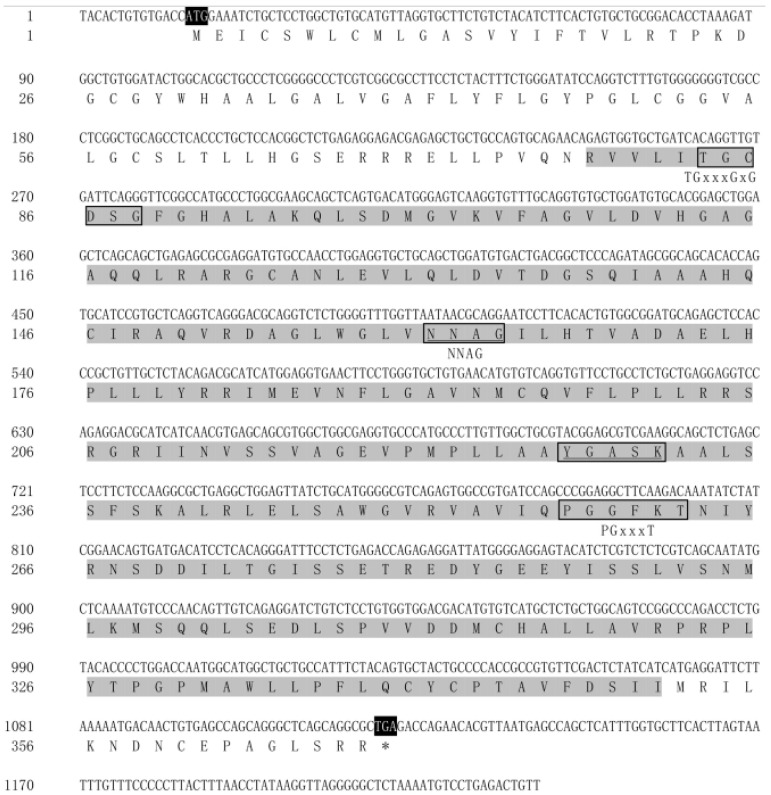
Identification of *hsd17b2* cDNA in *Monopterus albus.* The initiation codon of ATG and stop codon TGA are highlighted in black. The conserved structure of the SDR superfamily, i.e., the Rossmann-folding domain, is highlighted in gray. The classical binding motifs of 17 beta-HSD are in frames, namely TGxxxGxG, YxxxK, PGxxxT, and NNAG. * below the ‘TGA’ for the stop codon.

A 1230 bp fragment of *hsd17b2* cDNA was obtained from *M. albus* (named *Mahsd17b2*), containing 1107 bp of open reading frame and encoding 368 amino acids (aa) (Figure 1) with a molecular mass of 4.0 kDa. The predicted Hsd17b2 protein showed a Rossmann-folding domain, the highly conserved structure of the SDR superfamily, located at 78−351 aa; other putative binding motifs such as TGxxxGxG, YxxxK, PGxxxT, and NNAG were also found in the protein sequence.

**Figure 2:**
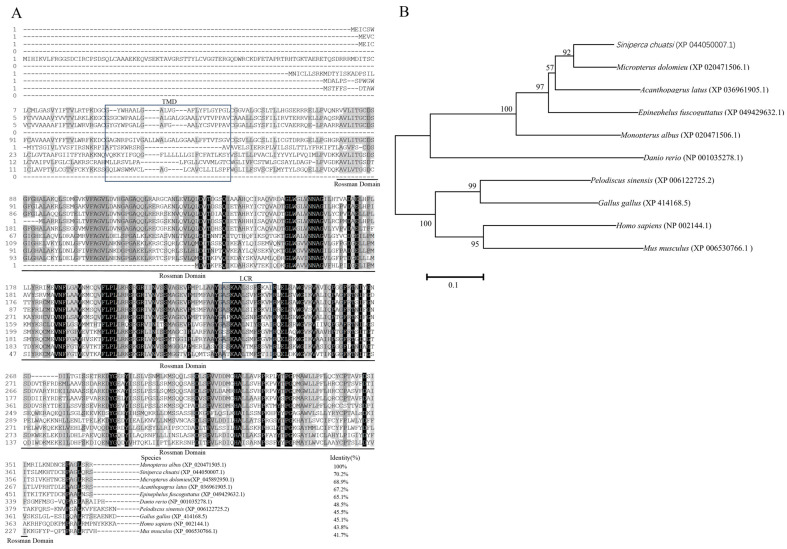
Homologous analysis and phylogenetic tree of Hsd17b2 proteins in *Monopterus albus.* (**A**) Multiple alignment of amino acid (aa) sequences of Hsd17b2 proteins. The protein sequences retrieved from NCBI (https://www.ncbi.nlm.nih.gov/, accessed on 16 May 2023), were aligned and calculated based on a ClustalW algorithm in DNAMAN (https://www.lynnon.com/dnaman.html, accessed on 16 May 2023). The amino acid sequence with 100% identity is highlighted in black, sequences with 75–100% identity are in gray, and sequences with 50% identity are in light gray. The TMD transmembrane region, the Rossmann-folding domain, and low complexity region are highlighted with black frames respectively. The species names and homology are listed at the end of the sequences. (**B**) Phylogenetic tree of Hsd17b2 proteins. The neighbor-joining phylogenetic tree of Hsd17b2 proteins was constructed by MEGA version 7 (https://www.megasoftware.net/, accessed on 16 May 2023), with a set of 1000 bootstraps in the neighbor-joining method. Scale bar tagged with 0.10 indicates the genetic distance, the number on each branch represents the bootstrap value.

Multiple alignment analysis showed that the sequences of Hsd17b2 proteins were poorly conserved across phyla owing to the diversity of amino acid sequences in the C-terminal (Figure 2A). Specifically, the identities of Hsd17b2 homologs among fish were less than 71%, and only around 45% aligned with mammalian Hsd17b2. By contrast, the Rossmann-folding domain, a nicotinamide cofactor binding motif in the N-terminal region, was well conserved in vertebrates. Phylogenetic tree analysis indicated that *M. albus* Hsd17b2 was clustered into the branch of Perciformes but separated from zebrafish, chicken, turtle, and mammals (Figure 2B).

**Figure 3:**
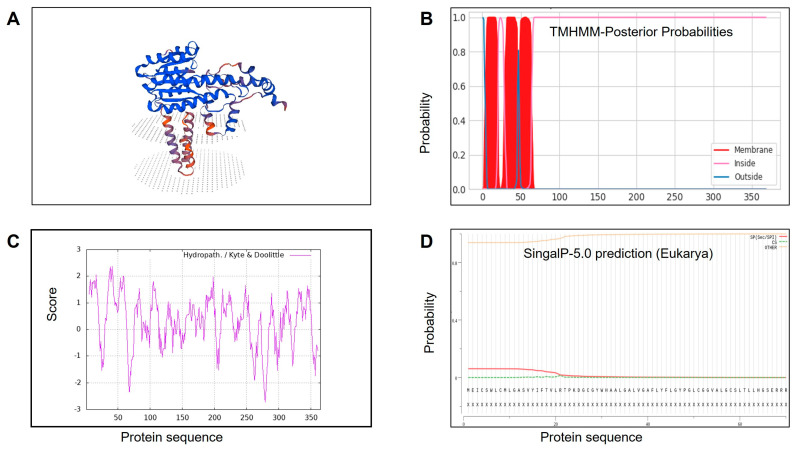
Bioinformatics analysis of Hsd17b2 protein in *Monopterus albus*. (**A**) Ribbon diagram of Hsd17b2 protein, containing α-helices and β-strands. α-helices are shown as coiled ribbons and β-strands as arrows; lines indicate random coils. (**B**) Predicted transmembrane region; the ordinate represents the probability of transmembrane transfer, and the abscissa indicates the sites of amino acid residues. (**C**) Hydrophilicity map of Hsd17b2 protein; the ordinate indicates the hydrophilicity index ranging from −2.711 to 2.367. (**D**) Signal peptide predicted by SignalP 5.0 (https://services.healthtech.dtu.dk/services/SignalP-5.0/, accessed on 16 May 2023). The probability of signal protein was about 5.98% and located at residues 1-21.

In addition, to further characterize the biochemical properties of Hsd17b2, a ribbon diagram of Hsd17b2 (Figure 3A) was constructed with α-helices and β-strands, dotted with random coils. Only one transmembrane region was calculated, ranging from 28 to 50 aa (Figure 3B). The predicted isoelectric point and instability index were computed to be 7.52 and 39.68, respectively. The grand average of hydropathicity was 0.311; hence, Hsd17b2 was classified as a hydrophobic protein (Figure 3C). Results of signal peptide prediction suggested only a 5.98% probability at the N-terminal region (Figure 3D), indicating that it may not be a secretory protein.

### 2.2. Expression Profiles of Mahsd17b2 mRNA in Ricefield Eel Tissues

**Figure 4:**
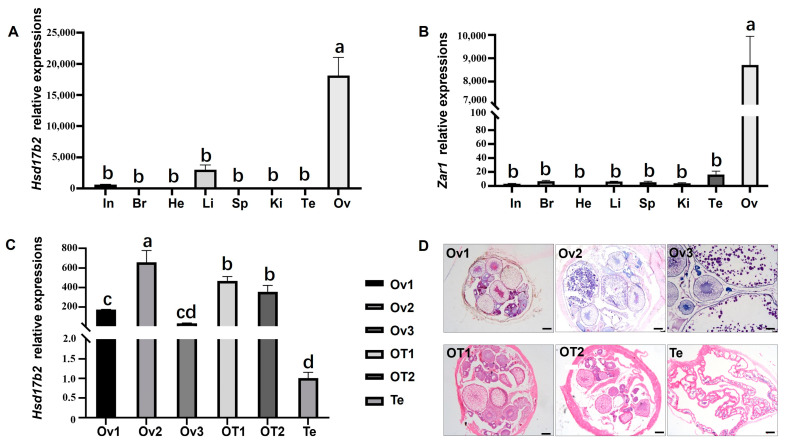
RT-qPCR analysis of *zar1* and *hsd17b2* mRNA in tissues. (**A**,**B**) Tissue-specific analysis of *zar1* and *hsd17b2* mRNA in adult *M. albus* tissues. A panel of tissues was collected and examined in this study, including the intestine (In), brain (Br), heart (He), liver (Li), spleen (Sp), kidney (Ki), testis (Te), and ovary (Ov). (**C**) Expression profiles of *hsd17b2* mRNA in gonads at different developmental stages. (**D**) Hematoxylin and eosin (HE) staining shows the structures of gonads at different developmental stages examined in this study. Ov1, ovaries from juvenile fish; Ov2, ovaries from young fish; Ov3, ovaries from adult fish; OT1, ovotestis at the early stage; OT2, ovotestis at the middle stage; Te, testis; scale bars, 200 μm. In (**A**–**C**), different letters (a–d) represent the significance between groups.

Real-time quantitative reverse transcription polymerase chain reaction (RT-qPCR) was conducted to examine the expression patterns of *Mazar1* and *Mahsd17b2* in various tissues and gonads at different developmental stages. As shown in Figure 4A, *hsd17b2* mRNA was highly expressed in ovary, weakly in liver and intestine, and scarcely in other tissues. Simultaneously, *zar1*, an oocyte-specific marker, was used as a control for analyzing the cellular distribution of *hsd17b2* mRNA (Figure 4B). Furthermore, *hsd17b2* mRNA exhibited significant dynamic expression in gonads at different developmental stages, which could be depicted as an M-shaped curve with two peaks: one in ovaries filled with oocytes at the vitellogenesis stage (Ov2, gonads from young fish) and the other in gonads of ovotestis at the early intersexual stage (OT1, gonads from mature fish), respectively (Figure 4C). In ovaries, the expression levels of *hsd17b2* first increased from Ov1 to Ov2, but dramatically dropped in Ov3. The second peak occurred in gonads at early intersexual stage, and then *hsd17b2* mRNA expression gradually declined in gonads at the intersexual stage and became undetectable in ricefield eel testis.

### 2.3. The Cellular Localization of hsd17b2 mRNA in Gonads

**Figure 5:**
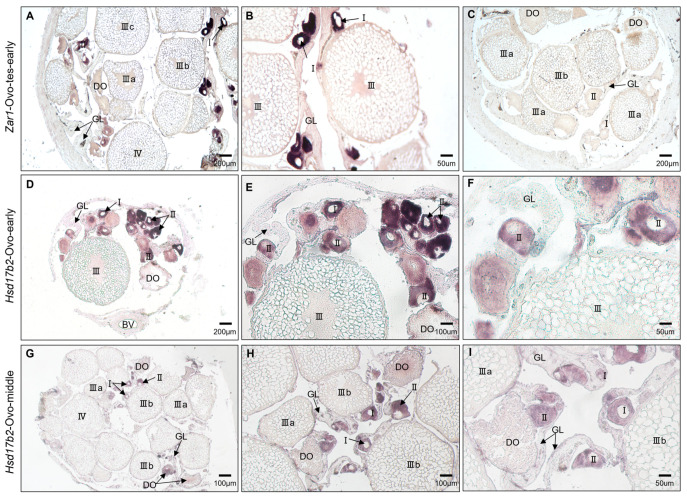
Cellular distribution of *zar1* and *hsd17b2* mRNA in ovaries. Chemical in situ hybridization was conducted on paraffin-embedded sections of ovaries. Antisense and sense RNA probes of *hsd17b2* gene were labeled with DIG; signals were developed with NBT/BCIP (in purple). (**A**–**C**) Ovaries from intersexual individuals; (**D**–**F**) ovaries from juvenile individuals; (**G**–**I**) ovaries from young fish. Cellular distribution results indicated that *hsd17b2* mRNA was strongly expressed in oogonia and then shifted into the somatic cells wrapping up the oocytes filled with yolk. The *zar1* gene specifically expressed in oocytes was used as the control for analyzing the cellular distribution of *hsd17b2* mRNA in the gonads of ovotestis at the early stage. I–IV, IIIa, IIIb, represents oocytes at different developmental stages; BV, blood vessels; DO, degenerated oocytes; GL, gonadal lamellae.

**Figure 6:**
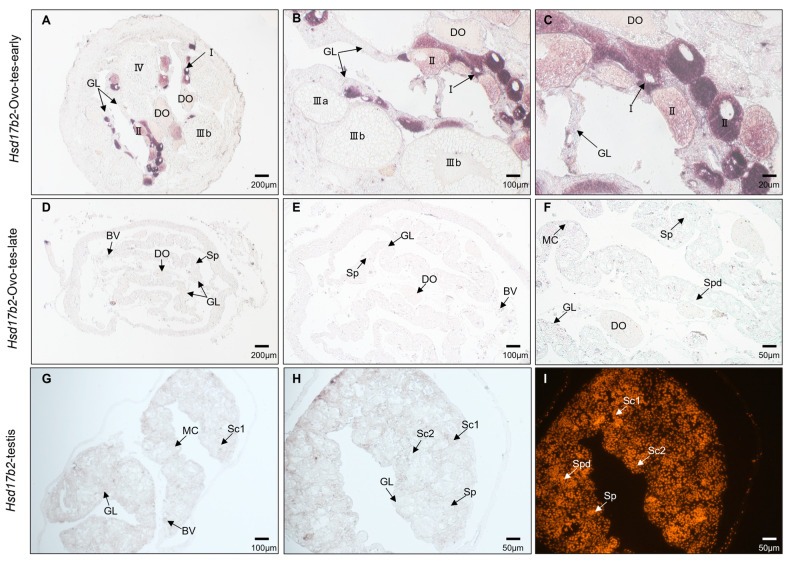
Cellular distribution of *hsd17b2* mRNA in ovotestis and testis. (**A**–**C**) Ovotestis at the early stage; (**D**–**F**) ovotestis at the late stage; (**G**–**I**) mature testis. The CISH showed that hsd17b2 mRNA expression patterns in gonads at the ovotestis stages were similar to those in ovaries. However, no signal was detected in the gonads of ovotestis at the late stage, and in testis, the testicular cell nuclei were counterstained by propidium iodide (PI, in red). I–IV, Ⅲa, Ⅲb represent oocytes at the different developmental stages. Sc1, primary spermatocyte; Sc2, secondary spermatocyte; Spd, spermatids; Sp, sperm; BV, blood vessels; DO, degenerated oocytes; GL, gonadal lamellae; MC, mesenchyme cluster.

To confirm the *hsd17b2* mRNA expression in gonadal cells, we examined the cellular distribution of *hsd17b2* mRNA by chemical in situ hybridization (CISH). Five typical stages were picked for CISH, including ovary at the early stage (Ovo-early), ovary at the developed stage (Ovo-middle); ovotestis at the early stage (Ovo-tes-early), ovotestis at the late stage (Ovo-tes-late), and testis (Te). The results showed that *hsd17b2* mRNA was mainly distributed in the cytoplasm of oogonia as well as the primary oocytes (Figure 5D,F). With the vitellogenesis of oocytes, cortisol alveoli accumulated in cytoplasm and follicular cells wrapped up the oocytes that were filled with yolk. The *hsd17b2* mRNA signals dispersed in a radiative shape from cytoplasm of the oocytes’ center to peripheral follicular cells (Figure 5G–I). In ovotestis at the early stage (Figure 6A–C), mesenchymal cells developed quickly with the thickness of gonadal lamellae, the degenerated oocytes and residual ovogonium were progressively surrounded and assimilated by mesenchymal clusters, and the peripheral theca cells and granulosa cells scaled up, which could be detected with weak *hsd17b2* mRNA signals. Intriguingly, *hsd17b2* mRNA signals gradually declined as oocytes developed and were almost diminished in the grown oocytes. Finally, the signals of *hsd17b2* mRNA were undetectable in gonads of ovotestis at the late stage (Ovo-tes-late) and testis (Figure 6D–I).

### 2.4. The hsd17b2 mRNA Expression in Gonads Regulated by Sex Hormone

**Figure 7:**
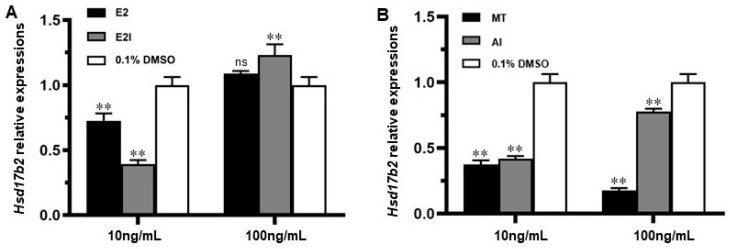
Effects of sex hormone treatment on *hsd17b2* mRNA expression in the *Monopterus albus* ovary. The ovaries of young fish were dissected and treated with 17β-estradiol (E2), 17β-estradiol inhibitor (E2I), melatonin (MT), and flutamide (androgen inhibitor, AI) respectively, and *hsd17b2* mRNA expression in the treated ovaries was examined by RT-qPCR. (**A**) Treatment with E2 and E2I at 10 ng/mL and 100 ng/mL. (**B**) MT and AI at 10 ng/mL and 100 ng/mL. The group treated with 0.1% DMSO was set as the control. Each treatment group was set up in triplicates, and the experiment was repeated twice. Data are shown as means ± SEM (n = 3). **, extremely significant (*p* < 0.01); ns, no significance *(p* > 0.05).

In order to determine how *hsd17b2* mRNA expression in gonad tissues is modulated by sex hormones treatment, fold-changes of gene expression were tested by RT-qPCR. In Figure 7A, compared to the control group, a 0.25-fold and 0.7-fold reduction (*p* < 0.01) were respectively observed from E2 and E2I treatments at a concentration level of 10 ng/mL. By contrast, the *hsd17b2* mRNA expression levels in the E2 treatment were close to the control while the E2I-treated group increased 0.1-fold (*p* < 0.01) at 100 ng/mL, which suggested that a higher concentration (100 ng/mL) of E2I may stimulate the *hsd17b2* mRNA expression. Moreover, the *hsd17b2* expression in gonads was significantly repressed by both MT and AI treatments (*p* < 0.01) (Figure 7B).

## 3. Materials and Methods

### 3.1. Animals and Ethics Statements

All ricefield eels in annual reproductive cycle were selected and purchased once every 2 months from a market (Rongchang, Chongqing, China), with 20–30 fish obtained each time. Within 2 reproductive years from January 2021 to December 2023, a total of 463 individuals (body length: 19.8–73.5 cm; body weight: 15.8–276.9 g) were collected. They were temporarily reared in an aquarium in the laboratory (water temperature 18 ± 2 °C) and then anesthetized with MS-222 (GUANYING Biotech, Wuhan, China). A panel of tissues were collected and stored at −80 °C for RNA extraction, including the intestines, brain, heart, liver, spleen, kidney, testes, and ovaries. Meanwhile, partial gonads were first fixed with 4% paraformaldehyde at room temperature for 12 h, and then dehydrated via a gradient alcohol series (70% alcohol (*v*/*v*), 80% alcohol (*v*/*v*), 90% alcohol (*v*/*v*), anhydrous alcohol) and stored at −20 °C for slicing. Stages of gonads were classified by histological analysis following hematoxylin and eosin (HE) staining. Animal experiments in this study were performed under the guidelines and approval of the Animal Care and Ethics Committee of Southwest University (Chongqing, China) (No. IACUC-20210111-01).

### 3.2. RNA Extraction and cDNA Synthesis

Total RNAs from different tissues were extracted using RNAiso Plus (Takara, Dalian, China). RNA integrity was determined by 1.0% agarose gel electrophoresis, and RNA was quantified by NanoDropOne (Thermo Fisher Scientific, Waltham, MA, USA). cDNA was then synthesized using a PrimeScript™ II 1st Strand cDNA Synthesis Kit (Takara, Dalian, China).

### 3.3. Cloning of the hsd17b2 cDNA Fragment

Specific primers for *hsd17b2* (Hsd17b2-F1/R1, Table 1) were designed by the NCBI Online designing tool Primer-BLAST (https://www.ncbi.nlm.nih.gov/tools/primer-blast/, accessed on 1 May 2023). The intersexual gonadal cDNAs were used as templates for gene cloning according to our preliminary experiments. Subsequently, PrimeSTAR^®^ HS (Premix) (Takara, Dalian, China) was used for amplification and the program was set to 98 °C for 30 s; 30 cycles of 60 °C for 30 s, 72 °C for 90 s; and 72 °C for 5 min. PCR products were examined by 1% agarose gel electrophoresis and then purified using a Tiangen gum recovery kit (TIANGEN BIOTECH, Beijing, China). pGEM^®^-T Easy vector (Promega, Madison, USA) and Top10 competent cells (TIANGEN BIOTECH, Beijing, China) were used for TA cloning. Finally, after the screening of colonies, the positive ones were picked up for sequencing (Tsingke Biotechnology company, Chongqing, China).

### 3.4. Sequence Analysis

The aa sequences of Hsd17b2 homologues in different species were retrieved from the NCBI database (https://www.ncbi.nlm.nih.gov/, accessed on 16 May 2023), and multiple sequence alignment was performed by DNAMAN (https://www.lynnon.com/dnaman.html, accessed on 16 May 2023). The phylogenetic tree was constructed using MEGA7.0 (https://www.megasoftware.net/, accessed on 16 May 2023) with the neighbor-joining (NJ) method (bootstrap = 1000). Moreover, CDD-search (https://www.ncbi.nlm.nih.gov/Structure/cdd/wrpsb.cgi, accessed on 16 May 2023), SWISS-MODEL (https://swissmodel.expasy.org/, accessed on 16 May 2023), SignalP 5.0 (https://services.healthtech.dtu.dk/services/SignalP-5.0/, accessed on 16 May 2023), THMM (https://services.healthtech.dtu.dk/services/TMHMM-2.0/, accessed on 16 May 2023), and Expasy-ProtScale (https://web.expasy.org/protscale/, accessed on 16 May 2023) were applied to analyze the conserved domain, ribbon diagram, signal peptide, transmembrane information, and hydrophilicity, respectively.

### 3.5. Gene Expression Analysis by RT-qPCR

To determine the expression patterns of *hsd17b2* mRNA in adult tissues, the intestine, brain, heart, liver, spleen, kidney, testis, and ovary were dissected and collected for RT-qPCR. Moreover, a panel of gonads at different developmental stages during sex reversal were collected, including ovaries at early stages from juvenile fish (Ov1), in which stage I–II oocytes were predominantly detected and tightly attached to the gonadal lamellae, and a few large oocytes with cortical alveolus were observed; ovaries at a more developed stage from young fish (Ov2), where stage III oocytes at vitellogenesis stages were closely arranged and almost filled in the whole gonadal cavity; and ovaries at a mature stage from adult fish (Ov3), where stage IV–V oocytes were full of yolk and about to be ovulated. Intersexual gonads included: ovotestis at the early stage of sex-reversal (OT1), numerous degenerated oocytes originating from oocytes at stage III–IV distributed in the gonadal cavity, together with thick gonadal lamellae; ovotestis at the middle stage of sex-reversal (OT2), in which gonadal lamellae gradually developed, the structure of testicular lobules as well as spermatogenesis were initiated, and more spermatocytes were obviously observed; testis from functional male individuals (Te), in which the testicular structure was well-developed, and mature sperm were clearly observed. In this study, Ov1, Ov2, and Ov3 were classified into pre-spawning stages, while OT1, OT2, and Te were in post-spawning stages, following the morphological criteria described in a previous report [28]. The gene-specific primers of *hsd17b2* and *zar1* were designed for RT-qPCR by Primer-BLAST (https://www.ncbi.nlm.nih.gov/tools/primer-blast/, accessed on 8 May 2023) and are listed in Table 1 (*Hsd17b2*-F2/R2, *Efla*-F/R, *Zar1*-F1/R1). *Ef1a* was used as the reference gene. TB Green^®^ Premix Ex TaqTII (Takara, Dalian, China) and QuantStudio 3 (Thermo Fisher Scientific, USA) were equipped for RT-qPCR, and the program was set up as 95 °C for 30 s; 40 cycles of 95 °C for 5 s, 60 °C for 34 s; and 95 °C for 15 s, 60 °C for 1 min, 95 °C for 15 s. The data were calculated with the 2^−ΔΔCt^ method, and the statistical analysis was conducted as a one-way ANOVA.

### 3.6. Chemical in Situ Hybridization (CISH)

Complementary RNA probes labeled by digoxin (DIG) of *M. albus hsd17b2* were prepared by the in vitro transcription method. Briefly, the *hsd17b2* cDNA fragment of 1230 bp was ligated into T-easy vector by A-T cloning (Promega, Madison, USA) and was then used as the template for PCR amplification. The cDNA fragments for RNA probes were amplified with the specific primer pairs (listed in Table 1, Hsd17b2-F3/R3, Zar1- F2/R2, T7, and SP6 promoter F/R, and then the probes were synthesized and purified using the DIG-probe kit following the manual (Roche Diagnostics GmbH, Mannheim, Germany). To prepare paraffin sections of gonads at different developmental stages for CISH, gonadal tissues were fixed in 4% paraformaldehyde (Sangon, Shanghai, China) at 4 °C overnight, and then gradually dehydrated by ethanol/PBS (60%, 80%, and 100%), then kept at −20 °C for 24 h. Gonad tissues were embedded in paraffin wax (SAKURA Tissue-Tek, Atlanta, GA, USA) and sectioned at 5 μm using a microtome (Themo Fisher, Wetzlar, Germany); CISH protocols were performed following the protocol described in a previous study [29].

### 3.7. Hormonal Treatment on Gonadal Tissues

Based on CISH and RT-qPCR analysis, it was found that the *hsd17b2* mRNA primarily expressed in the ovaries at the early developmental stages. Therefore, ovaries of 30 young females (mean body length: 27.2 ± 4.6 cm, mean body weight: 15.4 ± 3.8 g) were collected and cut into 1 mm^3^ pieces in DMEM medium (Gibco™, Grand Island, NE, USA) supplemented with 0.1 U/mL penicillin and 0.1 μg/mL streptomycin (Gibco™, Grand Island, NE, USA). Ovarian tissues were mixed thoroughly and divided equally into 1.5 mL tubes. After incubation in cell culture medium at 28 °C for 24 h, the medium was replaced and then treated with 10 ng/mL or 100 ng/mL of E2, melatonin (MT), and tamoxifen (17β-estradiol inhibitor, E2I). The 0.1% DMSO treatment was simultaneously set as the negative control. All treated samples were incubated for 24 h at 28 °C, each treatment was set in triplicate, and the in vitro experiments were repeated at least twice. Treated tissues were collected and then quickly frozen in liquid nitrogen and stored at −80 °C until use. To determine interactions between hormones and *hsd17b2*, the expression profiles of *hsd17b2* mRNA were examined in the treated tissues by RT-qPCR.

## 4. Discussion

### 4.1. Mahsd17b2 Gene Belongs to the 17-Beta Hydroxysteroid Dehydrogenase 2 Family

In this study, we cloned and characterized the *hsd17b2* cDNA fragment in *M. albus*, which belongs to the SDR superfamily [30] and contains putative binding motifs as well as conserved domains of the 17β-HSD family. Compared to mammals, MaHsd17b2 has conserved motifs YxxxK as the active site and TGxxxGxG involved in the Rossmann-fold domain as the cofactor binding [30]. However, sequence identity with mammals was much lower, with only 43.8% and 41.7% identity with human and mouse, respectively, owing to the great diversity in the amino acid sequence at the N-terminus. However, the N-terminal region of the HSD17B2 protein is usually considered as the region for endoplasmic reticulum localization without catalytic function in humans [31]. Our findings demonstrate that the sequence of Hsd17b2 exhibits considerable variation even among closely related fish, with no more than 70.2% identity, and less than 50% identity compared to zebrafish. This supports the hypothesis that the Hsd17b2 protein is functionally deficient in zebrafish [14] and suggests that such differences may have existed in the early evolution of fish. At the C-terminus, an obvious motif alteration from PRALR in mammals to EPAGL in fishes was found in our study. Notably, the motif PRALR was well-conserved in human, rat, and mouse, and is assumed to fasten the enzyme on the endoplasmic reticulum, play a role in translocation, and further constitute the full activity of the enzyme [31,32]. This transition might result in functional divergence of the Hsd17b2 protein in ricefield eel.

Advanced structure prediction showed a classical Rossmann-fold motif of the SDR superfamily composed of α-helices and β-strands accompanied by a conserved α/β sandwich folding pattern at the central position, which was similar to human [1]. MaHsd17b2 is a membrane-associated protein with a hydrophobic region at the N-terminus, and its hydropathy profile is identical to that of rat and catfish [33,34]. Unlike the insoluble property in humans, the hydrophilic lysine-rich motif (KKKAT) sequence at the C-terminus was absent in *M. albus*, which was similarly observed in mice and rats [32,35]. It was suggested that the hydrophilic lysine-rich motif may contribute to its full-length enzyme solubility. Moreover, no signal peptide was found in MaHsd17b2, being distinct from the human homolog exhibiting a type II signal anchor protein [31]. Although the feature of Hsd17b2 was poorly conserved across phyla, the Hsd17b2 enzyme of *M. albus* certainly belongs to the SDR superfamily, which regulates steroid metabolism based on the analysis of the MaHsd17b2 sequence and structure.

### 4.2. Mahsd17b2 mRNA Expressed in Ovaries at the Early Stage

*Hsd17b2* mRNA is widely detected in endometrium, placenta and other steroid-hormonal targeted tissues in mammals [6,32,35]. Here, *Mahsd17b2* mRNA was expressed highly in early stages of ovaries, poorly in liver and intestines, and rarely in other tissues, indicating that *hsd17b2* is predominantly expressed in ovary instead of testis. During the annual reproductive cycle, the expression profile of *hsd17b2* mRNA could be depicted as an M-shaped curve with two peaks in Ov2 (ovary at developed stages from young fish) and Ot1 (ovotestis at the early stage), and a dramatic decrease at Ov3 (ovary at the spawning stage) between Ov2 and Ot1. Variation in sex steroid levels necessarily impacts the expression of hormonal genes by acting on steroid response elements in promoters [13]. During the breeding season, similar expressional fluctuations in *hsd17b2* mRNA were observed in *Clarias magur* [33], suggesting that *hsd17b2* may be closely associated with the shift of E2 during the pre-spawning period, as expression of *hsd17b2* obviously increased from Ov1 to Ov2 to regulate oogenesis but decreased as spawning approached, when E2 was no longer needed for gonad development, which was responsible for changes in sex steroid levels in ricefield eel [36].

### 4.3. The Role of Mahsd17b2 in Maintaining the Balance of Sex Hormones in Ricefield Eel Gonads

Fish represent the earliest known vertebrate group to exhibit a range of distinct gonadal differentiation characteristics; the exogenous hormones, especially estrogens, can greatly influence the fate of both somatic and germ cells in gonads [37]. Numerous studies have reported successful induction of sex reversal through administration of exogenous hormones in gonochoristic species [37,38,39]. Generally, genetic and hormonal factors jointly act on sexual differentiation in ricefield eels. Sex steroid profiles have been observed throughout the sex-reversal process in ricefield eels [18,36]. Gonadal E2 stimulates oocyte development and is tightly involved in vitellogenesis of oocytes [37]. However, as spawning approaches, E2 will be replaced by other hormones such as 17α, 20β-dihydroxy-4-pregenen-3-one, and prostaglandin to induce the final maturation of oocytes [40]. Ovaries used in this experiment were in the pre-spawning period, so the level of E2 was likely close to the peak. We found that 100 ng/mL E2I treatment could stimulate *hsd17b2* mRNA expression, but no significant effect was found between E2 treatment and control. A possible explanation was that E2I bound to estrogen receptors, hence competitively inhibited the effect of E2, but T (the precursor of E2 that is catalyzed by aromatases) was continuously accumulated, and thus higher levels of T rather than E2 stimulated *hsd17b2* mRNA expression. Likewise, the AI treatment group significantly inhibited expression of *hsd17b2* mRNA in ricefield eels, and it has been reported that overexpression of *hsd17b2* in prostate cancer cell lines diminished androgen receptor signaling and suppressed androgen-induced cell proliferation [41]. The counteraction of AI led to a reduction in T, and thus may decrease *hsd17b2* mRNA expression.

Moreover, previous reports documented that androgenic precursors especially androstenedione, could rapidly accumulate during the spawning period. Androstenedione can be oxidated to T by the Hsd17b5 enzyme and can be simultaneously reduced from T by Hsd17b2 [13,41]. Intriguingly, the androstenedione level in ricefield eels showed a similar trend with the expression profile of *hsd17b2* mRNA in gonads before and after Ov3 (ovaries at developed stages) [36], which implies that hormonal transition may have existed before spawning and Hsd17b2 probably promotes this process.

MT participates in the development and maturation of gonads by acting on the hypothalamic pituitary gonadal axis. It can also directly act on oocytes to protect granulosa cells from antioxidation and regulate cellular autophagy, which is critical for oocyte maturation and reproductive quality [42]. MT synthesis in orange-spotted grouper (*Epinephelus coioides*) may coordinate with visual signals to induce gonadal sex change [43]. Furthermore, MT concentration obviously increased in male ricefield eels after sex reversal. Administration of MT in vivo suggested that it mainly functioned in gonads at pre-spawning stages. Appropriate doses of MT could stimulate the secretion of steroid hormones, while excessive doses of MT would suppress development of the gonads [44]. Similarly, in the in vitro treatment, the MT at both 10 ng/mL and 100 ng/mL significantly suppressed *hsd17b2* mRNA expression. As described in Section 2.3, we detected staining signals from degenerated oocytes in a regular shape, suggesting the potential relationship between *hsd17b2* mRNA expression and cellular autophagy. MT as an efficient reductant may decrease its expression by inhibiting cellular autophagy of granulosa cells.

Hormones regulate the expression of the *hsd17b2* gene in *M. albus*, which could give us an insight into the molecular mechanism of sex reversal. The expression of this gene could be regulated by E2 and E2I, and it plays a crucial role in maintaining the sex hormone balance. The initial surge of *hsd17b2* gene expression occurred at the early stage of ovarian development and then subsequently declined, which indicated that it functions in the transition from ovary to testis. Furthermore, the suppressive effects of MT and AI on *hsd17b2* expression may safeguard oocyte quality by regulating cellular autophagy. These findings elucidate the complex hormonal interplay governing sexual plasticity and provide a foundation for further investigations into the molecular mechanisms of sex reversal in vertebrates.

## 5. Conclusions

In conclusion, our findings highlight the *hsd17b2* gene structure, expression profiles, and feedback mechanisms under hormonal treatments in ricefield eels. The *Mahsd17b2* gene was closely involved in sex steroid metabolism and may promote the conversion of testosterone to androstenedione, thus contributing to the initiation of sex reversal. Our results also provide novel insight to understand the molecular mechanism of sex reversal in ricefield eels and a theoretical basis for extensive investigations of sex reversal in other hermaphroditic fishes.

## Figures and Tables

**Table 1:** The sequences of primer pairs used in this study.

Primer	Nucleotide Sequence	Tm (°C)	Product Length (bp)	Purpose
Hsd17b2-F1	5′-TACACTGTGTGACCATGGAAATC-3′	53.5	1237	For CDS cloning
Hsd17b2-R1	5′-AACAGTCTCAGGACATTTTAGAGC-3′	54.0		
Hsd17b2-F2	5′-GCAGAACAGAGTGGTGCTGA-3′	53.8	241	For RT-PCR and RT-qPCR
Hsd17b2-R2	5′-CAAACCCCAGAGACCTGCGT-3′	55.9		
EF1α -F	5′-CGCTGCTGTTTCCTTCGTCC-3′	59.7	102	
EF1α -R	5′-TTGCGTTCAATCTTCCATCCC-3′	55.5		
Zar1-F1	5′- GTGTGCGCTTTCAGTTCCTG-3′	54.5	163	
Zar1-R1	5′-ACACGGTACGGGTTGAAGTC-3′	58.8		
T7 promoter F	5′-TAATACGACTCACTATAGGG-3′	46.6	1315	For RNA Probe synthesis
Hsd17b2-R3	5′-AACAGTCTCAGGACATTTTAGAGC-3′	54.0		
T7 promoter F	5′-TAATACGACTCACTATAGGG-3′	46.6	807	
Zar1-R2	5′- AACGTGGCGTTGTGTTGTTG -3′	57.0		
Hsd17b2-F3	5′-TACACTGTGTGACCATGGAAATC-3′	53.5	1336	
SP6 promoter R	5′-ATTTAGGTGACACTATAGAAT-3′	44.0		
Zar1-F2	5′-AATCCCAAACTCACCCCGAG-3′	57.5	828	
SP6 promoter R	5′-ATTTAGGTGACACTATAGAAT-3′	44.0		

## Data Availability

All data have been included in the manuscript.

## Abbreviation

aa	Amino Acid
BV	Blood Vessels
cDNA	Complementary DNA
DIG	Digoxin
DO	Degenerated Oocytes
E2	17β-estradiol
E2I	Estradiol Inhibitor (Tamoxifen)
FC	Follicular Cell
GL	Gonadal Lamellas
HPG	Hypothalamic Pituitary Gonadal
Hsd17b2	17-Beta Hydroxysteroid Dehydrogenase 2
MT	Melatonin
AI	Androgen Inhibitor (Flutamide)
MC	Mesenchyme Cluster
NJ	Neighbor-Joining
OT1	Ovotestis at the Early Stage
OT2	Ovotestis at the Middle Stage
Ov1	Ovaries from Juvenile Fish
Ov2	Ovaries from Young Fish
Ov3	Ovaries from Adult Fish
Ovo-early	Ovary at the Early Stage
Ovo-medium	Ovary at the Developed Stage
Ovo-tes-early	Ovotestis at the Early Stage
Ovo-tes-late	Ovotestis at the Late Stage
RT-PCR	Reverse Transcription Polymerase Chain Reaction
RT-qPCR	Real-Time quantitative Polymerase Chain Reaction
Sc1	Primary Spermatocyte
Sc2	Secondary Spermatocyte
SDRs	Short-Chain Dehydrogenases/Reductases
Spm	Spermatids
Sp	Sperm
T	testosterone
Te	Testis
*Zar1*	Zygote Arrest-1

## References

[1] Kallberg Y., Oppermann U., Jörnvall H., Persson B. (2002). Short-chain dehydrogenases/reductases (SDRs). Eur. J. Biochem..

[2] Peltoketo H., Luu-The V., Simard J., Adamski J. (1999). 17beta-hydroxysteroid dehydrogenase (HSD)/17-ketosteroid reductase (KSR) family; nomenclature and main characteristics of the 17HSD/KSR enzymes. J. Mol. Endocrinol..

[3] Boucher E., Provost P., Plante J., Tremblay Y. (2009). Androgen receptor and 17beta-HSD type 2 regulation in neonatal mouse lung development. Mol. Cell Endocrinol..

[4] Labrie F., Luu-The V., Lin S., Simard J., Labrie C., El-Alfy M., Pelletier G., Bélanger A. (2000). Intracrinology: Role of the family of 17 beta-hydroxysteroid dehydrogenases in human physiology and disease. J. Mol. Endocrinol..

[5] Lukacik P., Kavanagh K., Oppermann U. (2006). Structure and function of human 17beta-hydroxysteroid dehydrogenases. Mol. Cell. Endocrinol..

[6] Miettinen M., Mustonen M., Poutanen M., Isomaa V., Vihko R. (1996). Human 17 beta-hydroxysteroid dehydrogenase type 1 and type 2 isoenzymes have opposite activities in cultured cells and characteristic cell- and tissue-specific expression. Biochem. J..

[7] Frycz B., Murawa D., Borejsza-Wysocki M., Marciniak R., Murawa P., Drews M., Jagodziński P. (2015). Expression of 17β-hydroxysteroid dehydrogenase type 2 is associated with some clinicopathological features in gastric cancer. Biomed. Pharmacother..

[8] Lee Y., He H., Shiue Y., Lee S., Lin L., Wu T., Chang I., Lee H., Li C. (2015). The prognostic impact of lipid biosynthesis-associated markers, HSD17B2 and HMGCS2, in rectal cancer treated with neoadjuvant concurrent chemoradiotherapy. Tumour Biol..

[9] Bulun S., Cheng Y., Pavone M., Yin P., Imir G., Utsunomiya H., Thung S., Xue Q., Marsh E., Tokunaga H. (2010). 17Beta-hydroxysteroid dehydrogenase-2 deficiency and progesterone resistance in endometriosis. Semin. Reprod. Med..

[10] Shen Z., Peng Z., Sun Y., Väänänen H., Poutanen M. (2008). Overexpression of human hydroxysteroid (17beta) dehydrogenase 2 induces disturbance in skeletal development in young male mice. J. Bone Miner. Res..

[11] Zhong Y., Rantakari P., Lamminen T., Toppari J., Poutanen M. (2007). Transgenic male mice expressing human hydroxysteroid dehydrogenase 2 indicate a role for the enzyme independent of its action on sex steroids. Endocrinology.

[12] Rantakari P., Strauss L., Kiviranta R., Lagerbohm H., Paviala J., Holopainen I., Vainio S., Pakarinen P., Poutanen M. (2008). Placenta Defects and Embryonic Lethality Resulting from Disruption of Mouse Hydroxysteroid (17-) Dehydrogenase 2 Gene. MolEndocrinol..

[13] Loveland J., Giraldo-Deck L., Kelly A. (2022). How inversion variants can shape neural circuitry: Insights from the three-morph mating tactics of ruffs. Front. Physiol..

[14] Mindnich R., Hrabe de Angelis M., Adamski J. (2007). Functional genome analysis indicates loss of 17beta-hydroxysteroid dehydrogenase type 2 enzyme in the zebrafish. J. Steroid Biochem. Mol. Biol..

[15] Baker M. (2004). Evolutionary analysis of 11beta-hydroxysteroid dehydrogenase-type 1, -type 2, -type 3 and 17beta-hydroxysteroid dehydrogenase-type 2 in fish. FEBS Lett..

[16] Zou C., Wang L., Zou Y., Wu Z., Wang W., Liang S., Wang L., You F. (2020). Characteristics and sex dimorphism of 17β-hydroxysteroid dehydrogenase family genes in the olive flounder *Paralichthys olivaceus*. J. Steroid Biochem. Mol. Biol..

[17] Cui H., Zhu H., Ban W., Li Y., Chen R., Li L., Zhang X., Chen K., Xu H. (2024). Characterization of two gonadal genes, zar1 and wt1b, in hermaphroditic fish Asian seabass (*Lates calcarifer*). Animals.

[18] Chan S., Phillips J. (1969). The biosynthesis of steroids by the gonads of the ricefield eel *Monopterus albus* at various phases during natural sex reversal. Gen. Comp. Endocrinol..

[19] Sheng Y., Chen B., Zhang L., Luo M., Cheng H., Zhou R. (2014). Identification of *dmrt* genes and their up-regulation during gonad transformation in the swamp eel (*Monopterus albus*). Mol. Biol. Rep..

[20] Yuan H. (2011). Effects of Different Exogenous Factors on Sex Reversal of *Monopterus albus*. Ph.D. Thesis.

[21] Zhu Y., Wang C., Chen X., Guan G. (2016). Identification of gonadal soma-derived factor involvement in *Monopterus albus* (protogynous rice field eel) sex change. Mol. Biol. Rep..

[22] Ruksana S., Pandit N., Nakamura M. (2010). Efficacy of exemestane, a new generation of aromatase inhibitor, on sex differentiation in a gonochoristic fish. Comp. Biochem. Physiol. Part C Toxicol. Pharmacol..

[23] Sun L., Jiang X., Xie Q., Yuan J., Huang B., Tao W., Zhou L., Nagahama Y., Wang D. (2014). Transdifferentiation of differentiated ovary into functional testis by long-term treatment of aromatase inhibitor in nile tilapia. Endocrinology.

[24] Tang F., Chan S., Lofts B. (1974). Effect of steroid hormones on the process of natural sex reversal in the rice-field eel, *Monopterus albus* (zuiew). Gen. Comp. Endocrinol..

[25] Hu Q., Guo W., Gao Y., Tang R., Li D. (2015). Molecular cloning and characterization of amh and dax1 genes and their expression during sex inversion in rice-field eel *Monopterus albus*. Sci. Rep..

[26] Liu J., Guiguen Y., Liu S. (2009). Aromatase (p450arom) and 11beta-hydroxylase (p45011beta) genes are differentially expressed during the sex change process of the protogynous rice field eel, *Monopterus albus*. Fish Physiol. Biochem..

[27] Zhang Y., Zhang W., Yang H., Zhou W., Hu C., Zhang L. (2008). Two cytochrome P450 aromatase genes in the hermaphrodite ricefield eel *Monopterus albus*: mRNA expression during ovarian development and sex change. J. Endorinol..

[28] Xiao Y., Liu Y. (1995). Study on the histology in sex changing from intersex to male of *Monopterus albus* (ZUIEW). J. Fish. China.

[29] Xu H., Gui J., Hong Y. (2005). Differential expression of vasa RNA and protein during spermatogenesis and oogenesis in the gibel carp (*Carassius auratus gibelio*), a bisexually and gynogenetically reproducing vertebrate. Dev. Dyn..

[30] Kavanagh K., Jörnvall H., Persson B., Oppermann U. (2008). Medium- and short-chain dehydrogenase/reductase gene and protein families: The SDR superfamily: Functional and structural diversity within a family of metabolic and regulatory enzymes. Cell. Mol. Life Sci..

[31] Wu L., Einstein M., Geissler W., Chan H., Elliston K., Andersson S. (1993). Expression cloning and characterization of human 17 beta-hydroxysteroid dehydrogenase type 2, a microsomal enzyme possessing 20 alpha-hydroxysteroid dehydrogenase activity. J. Biol. Chem..

[32] Akinola L., Poutanen M., Vihko R. (1996). Cloning of rat 17 beta-hydroxysteroid dehydrogenase type 2 and characterization of tissue distribution and catalytic activity of rat type 1 and type 2 enzymes. Endocrinology.

[33] Agarwal D., Gireesh-Babu P., Pavan-Kumar A., Koringa P., Joshi C., Gora A., Bhat I., Chaudhari A. (2020). Molecular characterization and expression profiling of 17-beta-hydroxysteroid dehydrogenase 2 and spermatogenesis associated protein 2 genes in endangered catfish, *Clarias magur* (Hamilton, 1822). Anim. Biotechnol..

[34] Casey M., MacDonald P., Andersson S. (1994). 17 beta-Hydroxysteroid dehydrogenase type 2: Chromosomal assignment progestin regulation of gene expression in human endometrium. J. Clin. Investig..

[35] Mustonen M., Poutanen M., Isomaa V., Vihko P., Vihko R. (1997). Cloning of mouse 17β-hydroxysteroid dehydrogenase type 2, and analysing expression of the mRNAs for types 1, 2, 3, 4 and 5 in mouse embryos and adult tissues. Biochem. J..

[36] Yeung W., Chan S. (1987). The plasma sex steroid profiles in the freshwater, sex-reversing teleost fish, *Monopterus albus* (zuiew). Gen. Comp. Endocrinol..

[37] Devlin R., Nagahama Y. (2002). Sex determination and sex differentiation in fish: An overview of genetic, physiological, and environmental influences. Aquaculture.

[38] Pandian T., Sheela S. (1995). Hormonal induction of sex reversal in fish. Aquaculture.

[39] Tenugu S., Senthilkumaran B. (2022). Sexual plasticity in bony fishes: Analyzing morphological to molecular changes of sex reversal. Aquac. Fish..

[40] Ogino Y., Miyagawa S., Iguchi T. (2016). Subchapter 94B. 17,20β-Dihydroxy-4-pregnen-3-one. Handbook of Hormone.

[41] Gao X., Dai C., Huang S., Tang J., Chen G., Li J., Zhu Z., Zhu X., Zhou S., Gao Y. (2019). Functional Silencing of *HSD17B2* in Prostate Cancer Promotes Disease Progression. Clin. Cancer Res..

[42] Cao Y., Shen M., Jiang Y. (2018). Melatonin reduces oxidative damage in mouse granulosa cells *via* restraining JNK-dependent autophagy. Reproduction.

[43] Li S., Li W., Jiang S., Jing Y., Xiao L., Yu Y., Liu Y., Li Y., Wang D., Li J. (2023). Mechanisms of sex differentiation and sex reversal in hermaphrodite fish as revealed by the *Epinephelus coioides* genome. Mol. Ecol. Resour..

[44] Shi Q., Lin H., Deng B. (1998). Effects of exogenous melatonin on gonadal development and secretion of gonadal hormones in the ricefield eel (*Monopterus albus*). Acta Zool.

